# Using an integrated social cognition model to predict COVID‐19 preventive behaviours

**DOI:** 10.1111/bjhp.12465

**Published:** 2020-08-11

**Authors:** Chung‐Ying Lin, Vida Imani, Nilofar Rajabi Majd, Zahra Ghasemi, Mark D. Griffiths, Kyra Hamilton, Martin S. Hagger, Amir H. Pakpour

**Affiliations:** ^1^ Department of Rehabilitation Sciences The Hong Kong Polytechnic University Hung Hom Hong Kong; ^2^ Pediatric Health Research Center Tabriz University of Medical Sciences Iran; ^3^ Social Determinants of Health Research Center Research Institute for Prevention of Non‐Communicable Diseases Qazvin University of Medical Sciences Iran; ^4^ International Gaming Research Unit Psychology Department Nottingham Trent University UK; ^5^ School of Applied Psychology Menzies Health Institute Queensland Griffith University Mt Gravatt Queensland Australia; ^6^ Psychological Sciences and Health Sciences Research Institute University of California, Merced California USA; ^7^ Faculty of Sport and Health Sciences University of Jyväskylä Finland; ^8^ Department of Nursing School of Health and Welfare Jönköping University Sweden

**Keywords:** attitude, behaviour change, intention, planning, preventive behaviours

## Abstract

**Objectives:**

Rates of novel coronavirus disease 2019 (COVID‐19) infections have rapidly increased worldwide and reached pandemic proportions. A suite of preventive behaviours have been recommended to minimize risk of COVID‐19 infection in the general population. The present study utilized an integrated social cognition model to explain COVID‐19 preventive behaviours in a sample from the Iranian general population.

**Design:**

The study adopted a three‐wave prospective correlational design.

**Methods:**

Members of the general public (*N* = 1,718, *M*_age_ = 33.34, *SD* = 15.77, male = 796, female = 922) agreed to participate in the study. Participants completed self‐report measures of demographic characteristics, intention, attitude, subjective norm, perceived behavioural control, and action self‐efficacy at an initial data collection occasion. One week later, participants completed self‐report measures of maintenance self‐efficacy, action planning and coping planning, and, a further week later, measures of COVID‐19 preventive behaviours. Hypothesized relationships among social cognition constructs and COVID‐19 preventive behaviours according to the proposed integrated model were estimated using structural equation modelling.

**Results:**

The proposed model fitted the data well according to multiple goodness‐of‐fit criteria. All proposed relationships among model constructs were statistically significant. The social cognition constructs with the largest effects on COVID‐19 preventive behaviours were coping planning (β = .575, *p *< .001) and action planning (β = .267, *p *< .001).

**Conclusions:**

Current findings may inform the development of behavioural interventions in health care contexts by identifying intervention targets. In particular, findings suggest targeting change in coping planning and action planning may be most effective in promoting participation in COVID‐19 preventive behaviours.

**Statement of contribution**

***What is already known on this subject?***

- Curbing COVID‐19 infections globally is vital to reduce severe cases and deaths in at‐risk groups.
- Preventive behaviours like handwashing and social distancing can stem contagion of the coronavirus.
- Identifying modifiable correlates of COVID‐19 preventive behaviours is needed to inform intervention.

***What does this study add?***

- An integrated model identified predictors of COVID‐19 preventive behaviours in Iranian residents.
- Prominent predictors were intentions, planning, self‐efficacy, and perceived behavioural control.
- Findings provide insight into potentially modifiable constructs that interventions can target.
- Research should examine if targeting these factors lead to changes in COVID‐19 behaviours over time.

## Background

Novel coronavirus disease 2019 (COVID‐19) infections, declared by the World Health Organization (WHO) as a pandemic (World Health Organization, 2020a), have had unprecedented global effects on people’s daily activities and way of life (Ahorsu, Lin, et al., 2020; Heymann & Shindo, 2020; Kobayashi *et al*., 2020; Lin, 2020; Pakpour, Griffiths, Chang, et al., 2020; Pakpour, Griffiths, & Lin, 2020a, 2020b; Tang *et al*., 2020). Despite government actions such as enforced self‐isolation, travel bans, and national lockdowns of non‐essential services, schools, and universities, infection and mortality rates continue to rise (Baud *et al*., 2020; Heymann & Shindo, 2020; Wu & McGoogan, 2020). Iran, as of 9 June 2020, is the tenth leading country in total reported cases of COVID‐19 and is continuing to experience a sharp rise in reported new cases of infections and deaths related to the infection: 175,927 total cases (+2,095 new cases) and 8,425 total deaths (+74 new deaths; Worldometer, 2020). To date, there is no vaccine to protect against COVID‐19 infection and therefore, non‐pharmacological interventions are the only currently available means to reduce the spread of infection and ‘flatten the curve’ of infection rates (Kim, Kim, Peck, & Jung, 2020). In response, the WHO has proposed a global action plan aimed at reducing the spread of COVID‐19 infections (World Health Organization, 2020b). The plan highlights the importance of adopting a range of health protection behaviours including, for example, washing hands frequently, maintaining social distancing, practising respiratory hygiene, and self‐isolating if feeling unwell (World Health Organization, 2020b). However, the WHO guidance is limited by the fact that it does not focus on understanding the mechanisms of action that underpin these preventive behaviours, or on strengthening individuals’ capacity, to adopt them.

Application of theories of social cognition has demonstrated promise in providing an understanding of the determinants of preventive behaviours (Hagger, Cameron, Hamilton, Hankonen, & Lintunen, 2020). Such theories help identify potentially modifiable factors that have been shown to be reliably related to behaviour. Once identified, these modifiable factors can inform the content and design of behavioural interventions aimed at promoting increased adherence to preventive behaviours in health contexts (Hagger, Cameron, et al., 2020; Kok *et al*., 2016). In the current study, we aimed to identify the key social psychological factors that underpin uptake and maintenance of the COVID‐19 preventive behaviours advocated by the WHO (World Health Organization, 2020b). We therefore focused on identifying the motivational and volitional determinants of COVID‐19 preventive behaviours among Iranians based on an integrated model of behaviour that combined social psychological constructs from the Theory of Planned Behavior (TPB; Ajzen, 1991; Ajzen & Schmidt, 2020) and the Health Action Process Approach (HAPA; Schwarzer, 2008; Schwarzer & Hamilton, 2020).

The TPB is a prominent social cognition theory that has been frequently applied to predict multiple health behaviours (McDermott *et al*., 2015; Rich, Brandes, Mullan, & Hagger, 2015). Intention is a focal construct of the theory and considered the most proximal predictor of behaviour. Intention is a function of three belief‐based constructs: attitudes (evaluation of the positive and negative consequences of the behaviour), subjective norms (perceived expectations of important others approving the intended behaviour), and perceived behavioural control (perceived capacity to carry out the behaviour). In addition, perceived behavioural control is proposed to directly predict behaviour when it closely approximates actual control. Although the extant literature applying the TPB has shown that intentions consistently predict health behaviour and mediate effects of the social cognition constructs on behaviour (Hagger, Chan, Protogerou, & Chatzisarantis, 2016; Hamilton, van Dongen, & Hagger, 2020; McEachan, Conner, Taylor, & Lawton, 2011; Rich *et al*., 2015), the intention–behaviour relationship is imperfect (Orbell & Sheeran, 1998; Rhodes & de Bruijn, 2013). Therefore, dual‐phase models of behaviour, such as the HAPA (Schwarzer, 2008; Schwarzer & Hamilton, 2020), propose a post‐intentional volitional phase in which individuals may employ a range of self‐regulatory strategies to enact their intentions.

One self‐regulatory strategy that may lead individuals to effectively enact on their intentions is planning. According to the HAPA, there are two types of planning: action planning and coping planning (Schwarzer, 2008; Schwarzer & Hamilton, 2020; Sniehotta, Schwarzer, Scholz, & Schüz, 2005). Action planning is a task‐facilitating strategy and relates to how individuals prepare themselves in performing a behaviour. This includes making plans of when, where, and how to perform the specific behaviour. Such plans connect the individual with good opportunities to act. Coping planning is a strategy that relates to how individuals prepare themselves in avoiding foreseen barriers and obstacles that may arise when performing a specific behaviour, and potentially competing behaviours that may derail the behaviour. Such plans protect good intentions from anticipated obstacles and competing behaviours.

Another important behavioural determinant proposed by the HAPA is self‐efficacy. In the HAPA, self‐efficacy is proposed to be important at all stages (i.e., motivational and volitional) of the health behaviour change process and is considered phase‐specific (Schwarzer & Hamilton, 2020; Zhang, Fang, Zhang, Hagger, & Hamilton, 2020; Zhang, Zhang, Schwarzer, & Hagger, 2019). Accordingly, several types of self‐efficacy can be distinguished: action self‐efficacy (an optimistic belief about personal agency during the pre‐actional, motivational phase) and maintenance self‐efficacy (an optimistic belief about personal agency during the post‐actional, volitional phase). Action self‐efficacy reflects individuals’ perceived capacity and confidence to engage in a behaviour in which they have not yet adopted or initiated (Schwarzer & Hamilton, 2020; Zhang et al., 2019, 2020). Maintenance self‐efficacy refers to individuals’ perceived confidence and ability in maintaining the behaviours they have already adopted and performed (Schwarzer & Hamilton, 2020; Zhang et al., 2019, 2020). Meta‐analytic research has provided support for the HAPA constructs of planning and self‐efficacy in predicting health behaviours (Zhang et al., 2019). Previous research has also shown intention, planning, and self‐efficacy to predict health preventive behaviours more specifically (Caudwell, Keech, Hamilton, Mullan, & Hagger, 2019; Cheng *et al*., 2019; Fung *et al*., 2019; Hamilton, Kirkpatrick, Rebar, & Hagger, 2017; Hou, Lin, Wang, Tseng, & Shu, 2020; Lin, Scheerma, Yaseri, Pakpour, & Webb, 2017; Lin *et al*., 2018, 2020; Lin, Updegraff, & Pakpour, 2016; Reyes Fernández, Knoll, Hamilton, & Schwarzer, 2016; Strong *et al*., 2018; Zhang *et al*., 2020).

### The current study

**Figure 1:**
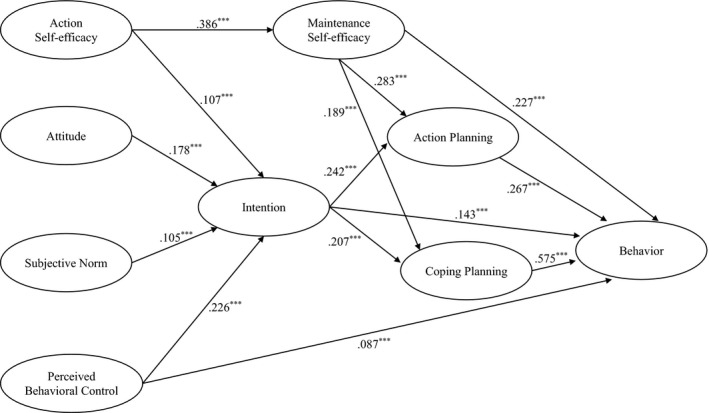
Standardized path coefficients among constructs from the integrated social cognition model for COVID‐19 preventive behaviours. Age sex, educational status, and occupational status were control variables in the model. *p < .05, **p < .01, ***p < .001.

Given the high rates of COVID‐19 infections worldwide, it is imperative that people engage in COVID‐19 preventive behaviours to ‘flatten the curve’ on rates of increase in new cases and, ultimately, reduce mortality rates from COVID‐19 infection. Identifying the key theory‐based determinants of key preventive behaviours (regular handwashing; respiratory hygiene practices; maintaining social distancing; self‐isolating) will help to inform effective interventions to promote participation in these behaviours. The purpose of the current study was to examine the efficacy of an integrated theoretical model of behaviour that incorporated constructs that represent motivational and volitional processes from the TPB and HAPA in predicting engagement in COVID‐19 preventive behaviours of Iranian individuals. The TPB and HAPA constructs of attitudes, subjective norms, perceived behavioural control, action self‐efficacy, and intention represented effects in the motivational phase of behavioural decision‐making. The HAPA constructs of maintenance self‐efficacy, action planning, and coping planning represented effects in the volitional phase of decision‐making. The study adopted a three‐wave correlational design with measures of constructs from the motivational phase taken at an initial data collection occasion (Time 1), constructs from the volitional phase taken at a first follow‐up occasion (Time 2), and measures of COVID‐19 preventive behaviours taken at a second follow‐up occasion (Time 3). Study hypotheses are outlined in the next section and illustrated in Figure 1.

The target behaviour selected in the current study was COVID‐19 preventive behaviours, which comprised four specific actions: regular handwashing, respiratory hygiene practices, maintaining social distancing, and self‐isolating. These behaviours all have the goal of preventing infection and spread of the virus in common and, therefore, have utility in attaining that goal. The proposed behavioural outcome, therefore, represents a behavioural category servicing a common goal. This is consistent with previous research examining the determinants of target behaviours that comprise multiple actions that service a particular goal. For example, researchers frequently aim to predict physical activity, which encompasses multiple actions (e.g., walking, cycling, swimming, running, going to the gym, playing various sports; Cheng *et al*., 2019; Fung *et al*., 2019; Rhodes & de Bruijn, 2013). We adopted a behavioural outcome comprising multiple actions in the current study because these behaviours have a common goal and may, therefore, have common determinants. Evidence for this comes from research examining the clustering of similar health behaviours, which demonstrates considerable consistency in the behaviours themselves and their determinants (e.g., Kremers, de Bruijn, Schalmaa, & Brug, 2004). Similarly, recent research has demonstrated that specific COVID‐19 preventive behaviours such as social distancing clusters with other health‐related behaviours such as physical activity (Bourassa, Sbarra, Caspi, & Moffitt, 2020). It is also important to note that although the determinants of the individual behaviours may differ at the level of the specific sets of beliefs that underpin the model constructs, when measuring the determinants at the global level, we expected the determinants to be consistent. Finally, we also expected consistency among the selected preventive behaviours and aimed to ensure this was the case by examining whether measures of the behaviours indicated a latent behavioural variable in our analyses.

In terms of specific model predictions, in the motivational phase of the proposed model, we expected that Time 1 attitudes, subjective norms, perceived behavioural control, and action self‐efficacy would be associated with Time 1 intentions. In addition, Time 1 intentions and perceived behavioural control were expected to predict Time 3 behaviour. It was also expected that Time 1 action self‐efficacy would predict Time 2 maintenance self‐efficacy. With respect to model relationships in the volitional phase, it was expected that Time 1 intentions would predict Time 2 action planning and coping planning, and Time 3 behaviour. Moreover, Time 2 maintenance self‐efficacy was expected to be associated with Time 2 action planning and coping planning. Finally, Time 2 maintenance self‐efficacy, action planning, and coping planning were expected to predict Time 3 behaviour.

A set of indirect effects consistent with theory was also specified. It was expected that Time 1 attitudes, subjective norms, and perceived behavioural control would predict Time 2 action planning and coping planning mediated by Time 1 intentions. In addition, attitudes, subjective norms, and perceived behavioural control were expected to predict Time 3 behaviour mediated by Time 1 intentions and Time 2 action planning and coping planning. We also expected Time 1 action self‐efficacy would predict Time 2 action planning and coping planning mediated by Time 1 intentions and Time 2 maintenance self‐efficacy. Additionally, we expected that Time 1 action self‐efficacy would predict Time 3 behaviour mediated by Time 1 intentions and Time 2 maintenance self‐efficacy, action planning, and coping planning. Finally, it was expected that Time 1 intentions and Time 2 maintenance self‐efficacy would predict Time 3 behaviour mediated Time 2 action and coping planning.

## Methods

### Participants and procedure

Participants were Iranian adults aged 18 years and older recruited via online social media platforms. We posted the web link to the survey on three popular social media sites in Iran: Instagram, Telegram, and WhatsApp. We also posted the link to several email listservs with many subscribers nationally. To be eligible for inclusion, participants had to be aged 18 years and older, had to provide consent to participate in the study, and had to have access to the Internet. The link directed respondents to an initial page describing study aims and requirements, followed by the consent form and, finally, the survey measures. Participants’ were prompted to provide their telephone number, email address, or social media contact details in order to receive a link to the follow‐up survey by SMS, email, or social media. Data were collected between 21 February 2020 and 17 March 2020. This period is critical to the immediacy of the current data as the first confirmed cases of COVID‐19 infections in Iran were reported on 19 February 2020 in Qom. By 21 February, 18 cases had been confirmed with a total death toll of four. Total confirmed cases had increased to 16,169 with 988 deaths by 17 March 2020. Media coverage of the pandemic was widely broadcast by state and private media during the period, with state broadcasters providing information on guidelines to prevent the spread of infection and social distancing rules. COVID‐19 hotlines were set up at the time to provide help and guidelines on COVID‐19 issues.

The study adopted a three‐wave correlational design with 1‐week intervals between each wave. Participants (*N* = 1,718; male = 796, female = 922) completed a survey at an initial data collection occasion (Time 1) comprising self‐report measures of action self‐efficacy, attitudes, subjective norms, perceived behavioural control, and intention. The survey also included self‐report measures of demographic factors including age, sex, education level, and employment status. At a second data collection occasion (Time 2), participants (*N* = 1,627, male = 760, female = 867, attrition rate = 5.30% from Time 1) completed self‐report measures of maintenance self‐efficacy, action planning, and coping planning. At a third data collection occasion (Time 3), participants (*N* = 1,569, male = 747, female = 849, attrition rate = 8.67% from Time 1) self‐reported their participation in COVID‐19 preventive behaviours performed over the past week. We conducted a statistical power analysis based on MacCallum, Browne, and Sugawara (1996) model fit criterion to establish the required sample size to detect effects. The analysis suggested a sample size of 1,456 was required for a well‐fitting model with an RMSEA of 0.05 against a null model with an RMSEA set at 0.00, 3 degrees of freedom, alpha set at 0.05, and power set at 0.80.

Data across each of the time points were matched using a code assigned to each participant. The study was conducted in accordance with the Declaration of Helsinki and was approved by the research ethics committee of BLINDED for review (Qazvin University of Medical Sciences (IR.QUMS.REC.1398.375)). All participants provided informed consent to participate prior to the first data collection occasion. Participants completing measures at all three data collection occasions received points valued at IRR 30,000 that were exchangeable for rewards. The points could be used to purchase healthy mobile phone apps like cognitive behavioural therapy, mindfulness, yoga, and weight management apps. Only those participants who completed all three surveys were rewarded.

### Measures

Psychological constructs were assessed on multi‐item psychometric instruments developed using standardized guidelines and adapted to make reference to the target behaviour in the current study, participation in COVID‐19 preventive behaviours. We collected data on different constructs across the three time points to allay common method variance and to provide prospective prediction of key outcomes in the integrated model over time. Brief details of the measures are provided below, and the full set of measures is available in Table 1. Questions were presented in Persian, a language commonly used and widely spoken in Iran. Current measures were adopted from those used in previous studies to tap TPB (Lin *et al*., 2016), phase‐specific self‐efficacy (Zhang *et al*., 2020), and planning (Strong *et al*., 2018) constructs.

**Table 1:** Items for all study measures with descriptive statistics, reliability coefficients, factor loadings, and average variance extracted statistics

Construct; Mean (SD)	Measurement item	λ	α	ω	CR	AVE
Attitude; 4.45 (0.62) Adapted from Lin *et al*. (2016)	*For me, following the recommendation of the WHO on engaging in preventive COVID‐19 behaviors every day in the coming week for me is …*		0.88	0.93	0.89	0.57
	extremely bad (1)/extremely good (5)	0.61				
	extremely undesirable (1)/extremely desirable (5)	0.70				
	extremely unenjoyable (1)/extremely enjoyable (5)	0.81				
	extremely foolish (1)/extremely wise (5)	0.88				
	extremely unfavorable (1)/extremely favorable (5)	0.76				
	extremely unpleasant (1)/extremely pleasant (5)	0.73				
Subjective norms; 3.87 (0.92) Adapted from Lin *et al*. (2016)	*Most people who are important to me would…*		0.77	0.77	0.77	0.62
	want me to perform the preventive COVID‐19 behaviors every day in the coming week	0.81				
	think I should perform the preventive COVID‐19 behaviors every day in the coming week	0.77				
Perceived Behavioural Control; 3.08 (1.14) Adapted from Lin *et al*. (2016)			0.90	0.92	0.90	0.75
	Whether or not I perform the preventive COVID‐19 behaviors every day in the coming week is completely up to me	0.85				
	I have resources, time and opportunities to perform the preventive COVID‐19 behaviors every day in the coming week	0.92				
	I am confident that if I want, I can perform the preventive COVID‐19 behaviors every day in the coming week	0.83				
Intention; 3.72 (1.09) Adapted from Lin *et al*. (2016)	*In the coming week, I…*		0.90	0.93	0.90	0.76
	am willing to perform the preventive COVID‐19 behaviors every day	0.82				
	want to perform the preventive COVID‐19 behaviors every day	0.94				
	plan to perform the preventive COVID‐19 behaviors every day	0.85				
Behaviour; 2.20 (0.69) Adapted from WHO. (2020b)	*How often do you perform the following behaviors as the WHO recommended…?*		0.80	0.84	0.80	0.51
	Regularly and thoroughly clean your hands with an alcohol‐based hand rub or wash them with soap and water	0.63				
	Practice respiratory hygiene (covering your mouth and nose with your bent elbow or tissue when you cough or sneeze)	0.76				
	Maintain at least one meter (3 feet) distance between yourself and anyone who is coughing or sneezing	0.80				
	Stay home if you feel unwell	0.65				
Action planning; 3.22 (0.97) Adapted from Strong et al. (2020)	*I have made a detailed plan regarding…*		0.83	0.86	0.83	0.62
	where to perform the preventive COVID‐19 behaviors every day	0.77				
	when to perform the preventive COVID‐19 behaviors every day	0.89				
	how to perform the preventive COVID‐19 behaviors every day	0.70				
Coping planning; 2.34 (1.09) Adapted from Strong et al. (2020)	*I have made a detailed plan regarding…*		0.89	0.79	0.86	0.68
	what to do if something interferes with my plans to perform the preventive COVID‐19 behaviors every day	0.84				
	how to cope with possible setbacks to perform the preventive COVID‐19 behaviors every day	0.87				
	what to do in difficult situations to act according to my intentions to perform the preventive COVID‐19 behaviors every day	0.75				
Action self‐efficacy; 3.27 (1.32) Adapted from Zhang *et al*. (2020)	*If you have not followed the recommendation of the WHO on the preventive COVID‐19 behaviors every day yet, do you have the confidence to start to follow the recommendation:*		0.88	0.88	0.88	0.71
	even if you have to force yourself to do so at the current stage	0.78				
	even if the planning for this is very laborious	0.92				
	even if you have to push yourself	0.83				
Maintenance self‐efficacy; 2.47 (1.09) Adapted from Zhang *et al*. (2020)	*If you are able to follow the recommendation of the WHO on the preventive COVID‐19 behaviors every day, do you have the confidence to maintain it in the long term:*		0.90	0.90	0.90	0.68
	even if you are stressed out	0.70				
	even if you feel tense	0.86				
	even if it takes you long to make it a habit	0.92				
	even if you are worried and troubled	0.81				

Note

λ = Standardized factor loading from structural equation model; α = Cronbach’s alpha reliability coefficient; ω = McDonald’s omega reliability coefficient; CR = composite reliability; AVE = average variance extracted from structural equation model.

#### Intention

Intention to perform the COVID‐19 preventive behaviours in the coming week was assessed using three items (e.g., ‘In the coming week, I am willing to perform the COVID‐19 preventive behaviors every day’), scored 1 = *strongly disagree* to 5 = *strongly agree*.

#### Attitude

Attitude was assessed using six semantic differential items in response to a common stem: ‘For me, following the recommendation of the WHO on engaging in COVID‐19 preventive behaviors every day in the coming week is…’. This was followed by a series of bipolar adjectives (e.g., *extremely bad*–*extremely good*). Responses were scored on five‐point scales.

#### Subjective norm

Subjective norm was assessed using two items measuring participants’ perceptions of their important others’ approval on performing the target behaviour (e.g., ‘Most people who are important to me would want me to perform the COVID‐19 preventive behaviors every day in the coming week’), scored 1 = *strongly disagree* to 5 = *strongly agree*.

#### Perceived behavioural control

Perceived behavioural control was assessed using three items measuring participants’ perceptions of their control and confidence in performing the target behaviour (e.g., ‘Whether or not I perform the COVID‐19 preventive behaviors every day in the coming week is completely up to me’), scored 1 = *strongly disagree* to 5 = *strongly agree*.

#### Action self‐efficacy

Action self‐efficacy was assessed using three items measuring participants’ perceived confidence in initiating the target behaviours immediately (e.g., ‘If you have not followed the recommendation of the WHO on the COVID‐19 preventive behaviors every day yet, do you have the confidence to start to follow the recommendation even if you have to force yourself doing so at the current stage’), scored 1 = *totally disagree* to 5 = *totally agree*.

#### Maintenance self‐efficacy

Maintenance self‐efficacy was assessed using four items measuring participants’ confidence in maintaining the target behaviour in the long term (e.g., ‘If you are able to follow the recommendation of the WHO on the COVID‐19 preventive behaviors every day, do you have the confidence to maintain it in the long term even if you are stressed out’), scored 1 = *totally disagree* to 5 = *totally agree*.

#### Action planning

Action planning was assessed using three items measuring the extent to which participants had made a plan in terms of how, when, and with whom to perform the target behaviour (e.g., ‘I have made a detailed plan regarding where to perform the COVID‐19 preventive behaviors every day’), scored 1 = *totally disagree* to 5 = *totally agree*.

#### Coping planning

Coping planning was assessed using three items measuring how much participants planned to overcome the obstacles preventing them from performing preventive behaviours (e.g., ‘I have made a detailed plan regarding what to do if something interferes with my plans’), scored 1 = *totally disagree* to 5 = *totally agree*.

#### Demographic variables

Participants self‐reported their age (in years), sex (coded as male = 1, female = 2), educational level (in years), and employment status (retired, homemaker, student, employed; coded as retired and homemaker = 1, student and employed = 2).

#### COVID‐19 preventive behaviour

Participants’ COVID‐19 preventive behaviour was assessed over the last week of the study. Participants reported their frequency of participation in four preventive behaviours recommended by the WHO: washing hands frequently, maintaining social distancing, practising respiratory hygiene, and staying home if feeling unwell (Ahorsu, Imani, et al., 2020; World Health Organization, 2020b; e.g., ‘Regularly and thoroughly clean your hands with an alcohol‐based hand rub or wash them with soap and water’). Before responding to the behavioural measure, participants were provided with a clear definition of the COVID‐19 preventive behaviours and recommendations for how and when they should be performed based on the WHO guidelines. Moreover, these guidelines corresponded with those provided by state media released by the Iranian Ministry of Health. Therefore, participants were fully aware of the definitions of the preventive behaviours and the guidelines. Responses to each behaviour were scored on five‐point scales (1 = *almost never* to 5 = *almost always*) and were used to indicate a latent COVID‐19 preventive behavioural variable in subsequent analyses. Higher scores indicated greater adherence to the WHO recommendations in engaging in COVID‐19 preventive behaviours.

### Data analysis

Hypothesized relationships among the proposed integrated social cognition model were analysed using structural equation modelling (SEM). The model was estimated using the AMOS software v24.0 with a maximum‐likelihood estimator and bias‐corrected bootstrapped standard errors approach with 5,000 resamples. Less than 10% of the data were missing and data were missing completely at random based on Little’s (1988) MCAR test (χ^2^ = 1.068, df = 4, *p* = .899). Missing data were imputed using the full information maximum‐likelihood method. Psychological and behavioural constructs were latent variables indicated by their respective sets of items. Hypotheses of the proposed integrated model were tested by specifying structural relationships between latent variables (see Figure 1), with each latent variable indicated by the set of scale items for each, including the behaviour factor. Age, sex, educational status, and employment status were included as non‐latent control variables in the model.

Overall model fit with the data was assessed using multiple fit indices: the goodness‐of‐fit chi‐square test, the comparative fit index (CFI), the Tucker–Lewis index (TLI), the standardized root‐mean‐square residual (SRMR), and the root‐mean‐square error of approximation (RMSEA). As the chi‐square test is highly oversensitive to even minor misspecification especially in large, complex models, values for the CFI and TLI that exceeded 0.95, and SRMR and RMSEA values that exceeded 0.05 and 0.06, respectively, were considered indicative of satisfactory fit of the model with the data (Hu & Bentler, 1999). Reliability of the study measures (intentions, attitudes, subjective norm, perceived behavioural control, action self‐efficacy, maintenance self‐efficacy, action planning, coping planning, and COVID‐19 preventive behaviours) was examined using either Cronbach’s α or McDonald’s ω coefficients and the composite reliability (CR) coefficient. Values for α and ω exceeding 0.70, and CR values exceeding 0.60, were considered indicative of adequate internal consistency. In addition, we also looked at the average variance extracted (AVE) for each latent variable to ensure that items were contributing adequately to the construct they indicated, with values in excess of 0.50 considered satisfactory.

The large sample size in the current study meant that most estimates of effects among study constructs in the SEM were likely to exceed conventional criteria for statistical significance (Ory & Mokhtarian, 2010; Wu, Chang, Chen, Wang, & Lin, 2015). As a consequence, assessment of effect sizes of parameter estimates among constructs from the proposed SEM was an imperative. Effect sizes were evaluated using standardized path coefficients, which allowed for the interpretation of absolute and relative effect sizes of the coefficients against Cohen’s suggested rules of thumb. Interpretation of effect sizes of standardized path coefficients for indirect effects was less easily interpretable as they comprised multiplicative composites of multiple effects. Based on previously suggested rules of thumb, we judged standardized path coefficients for indirect effects equal to or exceeding .075 as non‐trivial and effect sizes below this value as trivial (Hagger, Koch, Chatzisarantis, & Orbell, 2017; Seaton, Marsh, & Craven, 2010).

## Results

### Participant and attrition analysis

Demographic characteristics of participants who completed measures at each time point are presented in Table 2. Attrition analyses indicated that there were no significant differences in age (*F*(3,1,714) = 1.35; *p* = .26), gender distribution (χ^2^(3) = 2.77; *p* = .43), educational level (*F*(3,1,714) = 1.69; *p* = .17), employment status (χ^2^(3) = 3.23; *p* = .36), and psychological variables (Wilks’ λ = 1.00, *F*(8,1,618) = 0.68; *p* = .71), and preventive behaviours (*t*(1,594) = 0.20; *p* = .84) among participants who remained in the study at Time 3 and those who dropped out of the study at Time 1 or Time 2.

**Table 2:** Sample characteristics and descriptive statistics for study variables for participants at each stage of the study

Variable	Participants that completed surveys at all three time points (*N* = 1,563)	Participants that completed baseline and Time 2 surveys (*N* = 1,654)	Participants that completed baseline and Time 3 surveys (*N* = 1,658)	Participants that only completed the baseline survey (*N* = 1,660)
Age; *M* (*SD*)	33.60 (15.79)	31.25 (15.20)	29.42 (12.56)	30.84 (15.16)
Sex (female); *n* (%)	829 (48.25%)	884 (51.46%)	902 (52.50%)	887 (51.63%)
Educational level (in years); *M* (*SD*)	9.38 (4.28)	10.14 (4.06)	8.40 (3.90)	9.10 (4.21)
Employment status; *n* (%)				
Retired and housekeeper	449 (26.13%)	469 (27.30%)	476 (27.70%)	470 (27.36%)
Employed and student	1114 (64.84%)	1185 (68.98%)	1209 (70.38%)	1190 (69.27%)
Psychological variables; *M* (*SD*)				
Attitude at Time 1	4.44 (0.63)	4.57 (0.52)	4.56 (0.62)	4.46 (0.59)
Subjective norms at Time 1	3.86 (0.92)	3.90 (0.99)	4.20 (0.86)	3.80 (0.87)
Perceived behavioural control at Time 1	3.08 (1.15)	3.02 (1.06)	2.77 (1.21)	3.04 (1.11)
Intention at Time 1	3.72 (1.10)	3.90 (0.96)	3.87 (1.11)	3.74 (1.08)
Action planning at Time 2	3.21 (0.97)	3.34 (0.85)	NA	NA
Coping planning at Time 2	2.31 (1.10)	2.36 (0.78)	NA	NA
Action self‐efficacy at Time 1	3.22 (1.30)	3.44 (1.24)	1.35 (0.23)	3.10 (1.32)
Maintenance self‐efficacy at Time 2	2.48 (1.10)	2.53 (0.93)	NA	NA
Behaviour at Time 3; *M* (*SD*)	2.19 (0.70)	NA	2.17 (0.49)	NA

Note

*N* = 1,718. No statistically significant differences across groups were found on any of the descriptive statistics.

### Preliminary analysis

Descriptive statistics, reliability coefficients, factor loadings, and average variance extracted for study measures are presented in Table 1. Cronbach’s α and McDonald’s ω coefficients all exceeded 0.70, CR values were above 0.60, and all AVE values were above 0.50 supporting internal consistency and reliability of the measures. Consistent with the acceptable AVE values, factor loadings for each item on its respective latent factor was zero‐order factor correlations among study constructs are presented in Table 3. Most of the correlations were small to medium in effect size (i.e., *r* range .30 to .50), and all were statistically significant. Although each item representing a separate COVID‐19 preventive behaviour effectively indicated the latent behaviour variable, it was prudent to check the mean scores for each behaviour item to verify the consistency with which they were performed by participants. Mean scores were highly consistent (*M* range = 2.05 to 2.39) with high consistency in their variability (*SD* range = 0.85 to 0.89). A one‐way within‐participants ANOVA showed significant differences on each, which was unsurprising considering the large sample size. However, the small effect sizes for the differences (Cohen’s *d* range = 0.09 to 0.39) pointed to the consistency with which participants performed each behaviour, providing further justification for adopting a single COVID‐19 behaviour factor. Mean scores, standard deviations, mean differences, and test of difference for each preventive behaviour item are presented in Supplementary Table S1.

**Table 3:** Zero‐order latent variable correlations among study constructs

	1	2	3	4	5	6	7	8	9
1. Attitude	–								
2. Subjective norm	0.67**	–							
3. PBC	0.20**	0.09**	–						
4. Intention	0.33**	0.28**	0.30**	–					
5. Action planning	0.54**	0.54**	0.30**	0.28**	–				
6. Coping planning	0.26**	0.18**	0.40**	0.34**	0.36**	–			
7. Behaviour	0.49**	0.43**	0.44**	0.58**	0.59**	0.77**	–		
8. Action self‐efficacy	0.49**	0.54**	0.18**	0.27**	0.53**	0.30**	0.58**	–	
9. Maintenance self‐efficacy	0.33**	0.36**	0.10**	0.21**	0.34**	0.23**	0.47**	0.38**	–

Note

*N* = 1,718; PBC = perceived behavioural control; behaviour = COVID‐19 preventive behaviours.

* *p* < .01; **p < .001.

### Structural equation model

The integrated social cognition model proposed in the present study had good fit with the data (χ^2^ = 1,948.06, *df* = 497; *p* < .001; CFI = 0.957, TLI = 0.949, SRMR = .0822, RMSEA = .041, 90% CI = [0.039, 0.043]). Path coefficients for the direct effects among study constructs in the model are summarized in Figure 1 and path coefficients for the direct, indirect, and total effects are presented in Table 4. All proposed direct and indirect effects were statistically significant, although most effect sizes were small, with most of the standardized path coefficients less than .30. Perceived behavioural control had the largest effect on intention (β = .226, *p* < .001), with much smaller effects for action self‐efficacy, attitude, and subjective norms (βs < .178, *p*s < .001). The largest direct effects on COVID‐19 preventive behaviours were for coping planning (β = .575, *p* < .001), action planning (β = .267, *p* < .001), and maintenance self‐efficacy (β = .227, *p* < .001), while effects for intentions and perceived behavioural control were much smaller (βs < .143, *p*s < .001). Importantly, effects of intentions were mediated by both action planning and coping planning, consistent with the HAPA (total indirect effect, β = .194, *p* < .001), with a non‐trivial effect size, although a small residual effect of intention on behaviour (β = .143, *p* < .001). Along with the direct effect and the mediated effects through the planning constructs, there was also a total effect of intentions (β = .327, *p* < .001), again with a non‐trivial effect size. In addition, there were indirect effects of action self‐efficacy on behaviour through intentions, maintenance self‐efficacy, and the coping planning and action planning constructs (β = .194, *p* < .001). Furthermore, perceived behavioural control (β = .330, *p* < .001) and maintenance self‐efficacy (β = .411, *p* < .001) had the largest total effects on behaviour. The total effect of perceived behavioural control comprised a direct effect (β = .087, *p* < .001) and indirect effects through intention (β = .045, *p* < .001) and action planning and coping planning (β = .243, *p* < .001). The total effect of maintenance self‐efficacy comprised a direct effect (β = .227, *p* < .001) and indirect effects through the planning constructs (β = .184, *p* < .001).

**Table 4:** Direct, indirect, and total effects in the structural equation model testing relationships among the integrated social cognition model constructs for COVID‐19 preventive behaviours

Path	B (SE)	β	95% CI	
			LL	UL
Direct effects				
ASE* → *Intention	0.085 (0.025)	.107***	0.045	0.129
Attitude* → *Intention	0.272 (0.057)	.178***	0.169	0.374
SN* → *Intention	0.121 (0.050)	.105***	0.038	0.208
Perceived behavioural control* → *Intention	0.211 (0.024)	.226***	0.168	0.254
Perceived behavioural control* → *Behaviour	0.042 (0.010)	.087***	0.026	0.059
ASE* → *MSE	0.258 (0.019)	.386***	0.226	0.292
MSE → Action planning	0.295 (0.028)	.283***	0.244	0.347
MSE → Coping planning	0.232 (0.031)	.189***	0.177	0.285
MSE → Behaviour	0.140 (0.014)	.227***	0.115	0.166
Intention* → *Action planning	0.213 (0.023)	.242***	0.170	0.255
Intention* → *Coping planning	0.214 (0.027)	.207***	0.173	0.256
Intention* → *Behaviour	0.075 (0.011)	.143***	0.057	0.115
Action planning* → *Behaviour	0.159 (0.014)	.267***	0.137	0.183
Coping planning* → *Behaviour	0.290 (0.015)	.575***	0.264	0.318
Indirect effects				
ASE → Intention, MSE* → *Action planning	0.094 (0.013)	.135***	0.075	0.117
ASE → Intention, MSE* → *Coping planning	0.078 (0.012)	.095***	0.060	0.100
ASE → Intention, MSE → Action planning,coping planning → Behaviour	0.080 (0.010)	.194***	0.065	0.097
Attitude* → *Intention → Action planning	0.058 (0.016)	.043***	0.035	0.087
Attitude* → *Intention → Coping planning	0.058 (0.015)	.037***	0.036	0.086
Attitude* → *Intention → Behaviour	0.023 (0.006)	.029***	0.013	0.033
Attitude* → *Intention → Action planning, coping planning* → *Behaviour	0.046 (0.011)	.058***	0.029	0.066
Subjective norms* → *Intention → Action planning	0.026 (0.011)	.022**	0.009	0.047
Subjective norms* → *Intention → Coping planning	0.026 (0.012)	.025*	0.008	0.047
Subjective norms* → *Intention → Behaviour	0.010 (0.005)	.018*	0.003	0.019
Subjective norms* → *Intention → Action planning, coping planning* → *Behaviour	0.021 (0.009)	.034***	0.007	0.037
Perceived behavioural control → Intention → Action planning	0.045 (0.007)	.047***	0.035	0.059
Perceived behavioural control* → *Intention → Coping planning	0.045 (0.008)	.055***	0.032	0.060
Perceived behavioural control* → *Intention → Behaviour	0.021 (0.003)	.045***	0.016	0.027
Perceived behavioural control* → *Intention → Action planning, coping planning* → *Behaviour	0.118 (0.011)	.243***	0.101	0.136
Intention → Action planning, coping planning* → *Behaviour	0.096 (0.011)	.184***	0.079	0.114
MSE → Action planning, coping planning* → *Behaviour	0.114 (0.008)	.184***	0.093	0.137
Total effects				
ASE* → *Behaviour	0.080 (0.010)	.194***	0.065	0.097
Attitude* → *Behaviour	0.046 (0.011)	.058***	0.029	0.066
Subjective norms → Behaviour	0.021 (0.009)	.034***	0.007	0.037
Perceived behavioural control* → *Behaviour	0.161 (0.014)	.330***	0.138	0.185
Intention* → *Behaviour	0.171 (0.014)	.327***	0.148	0.194
MSE* → *Behaviour	0.255 (0.020)	.411***	0.222	0.289

Note

Age, sex, educational status, and occupational status were included as control variables in the structural equation model. AP = action planning; ASE = action self‐efficacy; B = unstandardized path coefficient; CP = coping planning; LL = lower limit of 95% CI; MSE = maintenance self‐efficacy; PBC = perceived behavioural control; SN = subjective norm; SE = standard error; β = standardized path coefficient; 95% CI = 95% confidence interval of unstandardized path coefficient; UL = upper limit of 95% CI.

*p < .05; **p < .01; ***p < .001.

Although the items of our COVID‐19 preventive behavioural measure effectively indicated the latent behaviour variable, for completion we also explored whether the effects in our model differed according to the specific preventive behaviour adopted as the target behaviour. We therefore re‐estimated our structural equation model with each of the four individual behaviours as the dependent variable, represented by single‐indicator latent variables. Results indicated high consistency in the pattern and size of the parameter estimates in each of the four models, and these were virtually unchanged from the estimates in the overall model. On the basis of these findings, our conclusions with respect to model effects remained unchanged (the analyses are summarized in Tables S2 to S4 and Figures S1–S4 in the supplemental materials).

## Discussion

The present study applied an integrated social cognition model to predict participation in COVID‐19 preventive behaviours among members of the Iranian general public. Findings lend support to the proposed relationships among the integrated social cognition model in identifying the determinants of COVID‐19 preventive behaviours. In particular, the research is consistent with previous studies applying the TPB and HAPA to identify the determinants of health behaviours and the processes involved (Hagger *et al*., 2016; McEachan *et al*., 2011; Rich *et al*., 2015; Zhang *et al*., 2019). The current model suggests that perceived behavioural control, intentions, forms of planning, and maintenance self‐efficacy are prominent behavioural determinants as they report non‐trivial indirect and total effects on COVID‐19 preventive behaviours. Current findings also support the importance of constructs representing both the motivational and volitional phases of action, again, consistent with previous research and syntheses of research applying the constituent theories (McEachan *et al*., 2011; Zhang *et al*., 2019). In particular, current findings support previous research applying these constructs to predict similar behaviours in other health‐related contexts, such as hand hygiene behaviours and face mask wearing (Contzen & Mosler, 2015; Zhang et al., 2019; Zomer *et al*., 2013), although the previous research was not conducted in the presence of a current pandemic while the current research was conducted at the peak of the ongoing COVID‐19 pandemic.

While the pattern of effects among model constructs in the current study was consistent with theory and identified salient determinants of COVID‐19 preventive behaviours, the majority of effects were small in magnitude. Even though the total effect of intentions on behaviour was non‐trivial, substantive variance in behaviour remained unexplained. Although shortfalls in the link between intention and behaviour are not uncommon in social cognition models (Orbell & Sheeran, 1998; Rhodes & de Bruijn, 2013), the link in the current study is particularly modest and suggests that individuals were not following through on their intentions to perform these preventive behaviours. This is aptly illustrated by the average levels of both variables in the current study, with the value for intentions (*M* = 3.73, *SD* = 1.10) exceeding the hypothetical midpoint on the five‐point scale and larger than the value for behaviour (*M* = 2.19, *SD* = 0.73), which was substantially below the midpoint. While it seems that coping planning and action planning accounted for a substantive proportion of the intention–behaviour relationship in the current study, results do not provide a sufficient explanation for the shortfall in the intention–behaviour relationship.

The apparent reluctance to engage in these in preventive behaviours is surprising given the high level of threat posed by the COVID‐19 outbreak in Iran and the widespread media coverage of the pandemic (Tuite *et al*., 2020). Furthermore, a recent study identified elevated levels of fear of COVID‐19 in the general Iranian population (Ahorsu, Lin, et al., 2020) and, although we did not assess risk perceptions in the current study, theory suggests that risk perceptions may translate into increased intentions to perform preventive behaviours to minimize risk (Rogers, 1975; Schwarzer, 2008; Schwarzer & Hamilton, 2020). However, one possible mitigating factor is that excessively heightened fear may be counterproductive in motivating individuals to engage in preventive behaviours (Lin, 2020). In fact, theory on illness beliefs and perceptions suggests that fear and beliefs reflecting high seriousness and consequences may motivate emotion‐focused coping responses aimed at mitigating fear, such as avoidance or denial, neither of which may be focused on behaviours to manage the risk itself (Hagger *et al*., 2017; Leventhal, Leventhal, & Contrada, 1998). This is also consistent with research demonstrating that heightened risk perceptions may not translate into performance of preventive behaviours when self‐efficacy is low (Peters, Ruiter, & Kok, 2013). However, these ideas remain speculative given we did not assess risk perceptions in the current study, and assessing risk perceptions and their interaction with self‐efficacy on performing preventive behaviours may be an important avenue for future research.

It is also important to consider possible contextual influences on the low COVID‐19‐related behavioural response and modest intention–behaviour relationship in the current study. The study was conducted in the run‐up to the Persian New Year on 3 March 2020. Consequently, many Iranians may have been reluctant to follow COVID‐19 preventive behaviours and resisted government and WHO recommendations. Traditional New Year’s celebrations in Iran involve large family gatherings and social events, festive behaviours that are ingrained and habitual, and form a strong part of the Persian culture. Given the cultural significance of this celebration, it is possible that the traditional festive behaviours may have taken precedence over performing COVID‐19 preventive behaviours, particularly the social distancing aspect, as they are incompatible.

Modest effect sizes among model constructs notwithstanding, the current study is among the first to provide preliminary evidence of the potentially modifiable constructs that relate to preventive behaviours known to be critical in minimizing the spread of COVID‐19 infections. Current findings may contribute to efforts to increase population‐level participation in preventive behaviours by signposting the constructs that should be targeted in behavioural interventions. Research that identifies constructs that are reliably related to behaviour form an important part of the process by which interventionists develop behavioural interventions (Hagger, Moyers, McAnally, & McKinley, 2020; Rothman, Klein, & Sheeran, 2020). This can be coupled with recent research that has linked these constructs with sets of methods or *techniques* purported to change them based on theory and previous evidence. Interventionists can therefore identify appropriate techniques that may be effective in affecting change in the behaviour of interest by targeting change in the target constructs, a *mechanism of action* (Connell *et al*., 2018). The current study, therefore, may provide part of the chain of evidence necessary to develop effective behaviour change interventions for COVID‐19 preventive behaviours.

Based on current evidence, interventionists should consider strategies that target change in perceived behavioural control, action and maintenance self‐efficacy, and coping planning as these the constructs had the largest direct and indirect effects on COVID‐19 preventive behaviour. Strategies known to promote self‐efficacy include providing opportunities to experience success with the behaviour through, for example, demonstration, modelling, and positive feedback (Warner & French, 2020). These strategies could be tailored to focus on uptake of the behaviour in the motivational (e.g., demonstrating what is an appropriate social distance when waiting in line at a grocery store; showing effective handwashing technique and prompting practice) or maintenance (e.g., prompting individuals to identify an appropriate rule of thumb on keeping an appropriate social distance every time one is in a store; how to incorporate handwashing into a daily routine) phase. Similarly, promoting effective coping planning entails prompting individuals to identify potential barriers to the target behaviour and identifying potential actions that can be put in place to mitigate them (e.g., for the barrier of not having access to handwashing facilities, an individual could plan to make sure they have a personal supply of alcohol‐based hand sanitizer available; Rhodes, Grant, & de Bruijn, 2020). These strategies would form the content of communications delivered through various media (e.g., television, leaflets, posters, web‐based messages) to the affected population.

### Strengths, limitations, and recommendations for future research

The current research has a number of strengths: (1) identifying the determinants of a set of appropriate behaviours aimed at preventing spread of COVID‐19, an infection that poses a substantive global health threat and a priority area for behavioural intervention; (2) adoption of an appropriate integrated theoretical model that provides a set of a priori predictions on the motivational and volitional determinants of COVID‐19 preventive behaviours; (3) recruitment of a large sample of participants in a population subjected to substantive threat of infection; and (4) use of appropriate longitudinal study design, previously validated measures, data collection techniques, and analytic methods. However, a number of limitations to the current data should be noted. First, although the prospective design provides some basis for the temporal order of relationships among constructs, the current data are correlational, so inferences of causality were drawn from theory alone and not the data. Furthermore, the prospective design did not model the covariance stability or change in constructs over time. This is an important caveat to consider when making recommendations for practice. While correlations between constructs and behavioural outcomes may provide some indication of potential targets for intervention, these data do not provide sufficient basis that affecting change in a construct will lead to change in a behavioural outcome, future research adopting panel designs that model change in constructs over time, and intervention or experimental designs that affect change constructs and observe their effects on behavioural outcomes, are needed.

It is also important to note that the study was conducted over 2‐week period, a relatively brief follow‐up period. The short time period is appropriate given the high speed of transmission of the coronavirus, creating an imperative for immediate mass adoption of COVID‐19 preventive behaviours in the population to prevent widespread infection. However, the current study does not provide evidence on the extent to which model constructs predict COVID‐19 preventive behaviours over a longer period, and long‐term follow‐up would provide important data on long‐term maintenance of these behaviours. Moreover, it is important to note that the current study relied exclusively on self‐report measures. Although we adopted previously validated measures which demonstrated good reliability and construct validity, such measures have the potential to introduce error variance through recall bias and socially desirable responding. Future studies may consider verification of behavioural data with non‐self‐report data such as data on infection rates.

Another important limitation is the aggregation of multiple COVID‐19 preventive behaviours into a single behavioural score representing COVID‐19 preventive behaviours with corresponding social cognition measures that made reference to those specific behaviours rather than the general category of COVID‐19 preventive behaviours. Our original rationale for this was that these behaviours all service the same goal and, therefore, we would expect these behaviours to be closely aligned and therefore, have the same determinants and the same strength of effects within the proposed model. Evidence for this comes from the high factor loadings of each behavioural measure on the latent COVID‐19 preventive behaviours variable, suggesting relative consistency in the way participants’ performed these behaviours. In addition, estimation of the model with each of the behavioural items as the target behaviour demonstrated substantive consistency in model effects. Taken together, these findings provide evidence that the pattern and size of model effects observed in the current study are consistent across the behaviours. Nevertheless, we cannot unequivocally rule out idiosyncratic variation in the determinants, and the strength of their effects, on the model constructs for each specific preventive behaviour. This could only be done by examining the corresponding determinants of each specific behaviour separately and then testing the invariance of the model effects for the model for each behaviour. This remains an imperative for future research.

In addition, the stem phrases used in the items might have presented difficulties for some participants to interpret the item meaning. For example, the self‐efficacy items were prefixed with the phrase: ‘If you are doing….’, and other items included the prefix: ‘If you have not followed the recommendation of the WHO…’. Participants without such experience or had followed recommendations might have had difficulties understanding the item content. Finally, some of the items aimed at assessing COVID‐19 preventive behaviours might have been difficult for people to answer. Problems with interpreting these items for some participants may have introduced additional error variance to the measures and, therefore, affected the strength of model relationships involving these variables.

It is also important to acknowledge that we cannot confirm that the current model was conducted in a context where individuals were adopting the COVID‐19 behaviours for the first time. The current research was conducted during a period when it is likely that participants were just starting to introduce these new behaviours given that very few cases of COVID‐19 had been detected in Iran at the time. Nevertheless, we cannot unequivocally rule out that a proportion of the participants were not already enacting these behaviours, or rule out the possibility that some participants already had substantive experience with these behaviours, albeit with a different goal. So, while it is likely that the current research captured individuals when they were adopting behaviours for the first time, we cannot rule out the possibility of past experience with the behaviours. Future research should consider the inclusion of past behaviour as an additional predictor in the model consistent with previous research applying social cognition theories (e.g., Brown, Hagger, & Hamilton, 2020; Chatzisarantis, Hagger, Smith, & Phoenix, 2004; Hagger *et al*., 2016; Hagger, Polet, & Lintunen, 2018).

### Conclusion

Urgent action is required to stem the spread of COVID‐19 in order to ‘flatten the curve’ of infection rates and minimize stress on available resources and health care facilities, and, importantly, reduce mortality. The current study identified a number of important social psychological determinants of participation in COVID‐19 preventive behaviours, particularly forms of self‐efficacy, perceived behavioural control, and planning. Assuming these determinants are modifiable through intervention, the current research provides important formative data that may assist development of optimally effective behavioural interventions. However, the relatively low levels of participation in these preventive behaviours endemic in the current population are a concern. Future research should consider testing the efficacy of behavioural interventions that target change in the constructs identified in the current study using appropriately matched behaviour change techniques. In addition, longitudinal studies adopting panel designs are also a priority to identify directional effects among theory constructs in this high priority context.

## Funding

This study was supported by grants from the Qazvin University of Medical Sciences. The funders were not involved in the study design, data collection, data analysis, data interpretation, or writing of the report. Martin S. Hagger’s contribution was supported by a Finland Distinguished Professor (FiDiPro) award (#1801/31/2105) from Business Finland.

## Conflicts of interest

The authors declare that they have no competing interests.

## Ethical approval

The study was approved by the ethics committee of the Qazvin University of Medical Sciences (IR.QUMS.REC.1398.375). Participants completed an online informed consent form before beginning the survey.

## Consent for publication

Not applicable.

## Author contributions

All authors contributed to the study design, data interpretation, editing, and critical review of the manuscript. VI, NRM, and Z Gh collected data. AHP and CYL performed the data handling and data analysis and drafted the first manuscript. MDG interpreted the data and its analysis. KH and MSH helped draft and revise the manuscript. All authors read and approved the final manuscript.

## Supporting information


**Table S1**. Comparisons of Mean Scores on the Four Preventive Behavior Items
**Table S2**. Direct, Indirect, and Total Effects in the Structural Equation Model Testing Relations Among the Integrated Social Cognition Model Constructs with Hand Hygiene as the Target Behavior
**Table S3**. Direct, Indirect, and Total Effects in the Structural Equation Model Testing Relations Among the Integrated Social Cognition Model Constructs with Practicing Respiratory Hygiene as the Target Behavior
**Table S4**. Direct, Indirect, and Total Effects in the Structural Equation Model Testing Relations Among the Integrated Social Cognition Model Constructs with Maintaining a One‐Meter Distance as the Target Behavior
**Table S5**. Direct, Indirect, and Total Effects in the Structural Equation Model Testing Relations Among the Integrated Social Cognition Model Constructs with Staying at Home if Unwell as the Target Behavior
**Figure S1**. Standardized path coefficients among constructs from the integrated social cognition model with hand hygiene as the target behavior.
**Figure S2**. Standardized path coefficients among constructs from the integrated social cognition model with practicing respiratory hygiene as the target behaviour
**Figure S3**. Standardized path coefficients among constructs from the integrated social cognition model with maintaining a one meter distance as the target behavior
**Figure S4**. Standardized path coefficients among constructs from the integrated social cognition model with staying at home if unwell as the target behaviour.
