# Supplementary material for: Using an integrated social cognition model to predict COVID‐19 preventive behaviours
Source: Br J Health Psychol. 2020 Aug 11;25(4):981–1005. doi: 10.1111/bjhp.12465 (PMC7436576; doi:10.1111/bjhp.12465)
Supplement: Supplementary file 1 — Table S1. Comparisons of Mean Scores on the Four Preventive Behavior Items Table S2. Direct, Indirect, and Total Effects in the Structural Equation Model Testing Relations Among the Integrated Social Cognition Model Constructs with Hand Hygiene as the Target Behavior Table S3. Direct, Indirect, and Total Effects in the Structural Equation Model Testing Relations Among the Integrated Social Cognition Model Constructs with Practicing Respiratory Hygiene as the Target Behavior Table S4. Direct, Indirect, and Total Effects in the Structural Equation Model Testing Relations Among the Integrated Social Cognition Model Constructs with Maintaining a One‐Meter Distance as the Target Behavior Table S5. Direct, Indirect, and Total Effects in the Structural Equation Model Testing Relations Among the Integrated Social Cognition Model Constructs with Staying at Home if Unwell as the Target Behavior Figure S1. Standardized path coefficients among constructs from the integrated social cognition model with hand hygiene as the target behavior. Figure S2. Standardized path coefficients among constructs from the integrated social cognition model with practicing respiratory hygiene as the target behaviour Figure S3. Standardized path coefficients among constructs from the integrated social cognition model with maintaining a one meter distance as the target behavior Figure S4. Standardized path coefficients among constructs from the integrated social cognition model with staying at home if unwell as the target behaviour. [file BJHP-25-981-s001.docx]

Table S1.

*Comparisons of Mean Scores on the Four Preventive Behavior Items*

| Item | Mean (SD) | Mean difference between items (*d*) | | | |
| --- | --- | --- | --- | --- | --- |
|  |  | Item 1 | Item 2 | Item 3 | Item 4 |
| 1: Clean hand or washing | 2.39 (0.89) | – |  |  |  |
| 2: Practicing respiratory hygiene | 2.13 (0.89) | 0.266 (0.29) ^***^ | – |  |  |
| 3: Maintaining at least one meter | 2.05 (0.85) | 0.345 (0.39) ^***^ | 0.079 (0.09) ^***^ | – |  |
| 4: Staying at home if you feel unwell | 2.22 (0.86) | 0.169 (0.19) ^***^ | -0.097 (0.10) ^***^ | -0.176 (0.20) ^***^ | – |

*Note.* *d* = Cohen’s *d* effect size estimate

^*^*p* < .05, ^**^*p* < .01, ^***^*p* < .001

Table S2

*Direct, Indirect, and Total Effects in the Structural Equation Model Testing Relations Among the Integrated Social Cognition Model Constructs with Hand Hygiene as the Target Behavior*

| Path | B (SE) | β | 95% CI | |
| --- | --- | --- | --- | --- |
|  |  |  | LL | UL |
| Direct effects |  |  |  |  |
| ASE*→*Intention | 0.082(0.025) | 0.104*** | 0.042 | 0.125 |
| Attitude*→*Intention | 0.270(0.062) | 0.177*** | 0.167 | 0.372 |
| SN*→*Intention | 0.121(0.051) | 0.105* | 0.038 | 0.208 |
| Perceived behavioral control*→*Intention | 0.212(0.026) | 0.227*** | 0.169 | 0.255 |
| Perceived behavioral control*→*Behavior | 0.048(0.019) | 0.060* | 0.017 | 0.079 |
| ASE*→*MSE | 0.253(0.020) | 0.380*** | 0.221 | 0.287 |
| MSE→Action planning | 0.295(0.031) | 0.282*** | 0.244 | 0.348 |
| MSE→Coping planning | 0.232(0.033) | 0.189*** | 0.177 | 0.286 |
| MSE→Behavior | 0.150(0.026) | 0.146*** | 0.107 | 0.192 |
| Intention*→*Action planning | 0.212(0.026) | 0.241*** | 0.170 | 0.255 |
| Intention*→*Coping planning | 0.213(0.026) | 0.206*** | 0.172 | 0.256 |
| Intention*→*Behavior | 0.055(0.019) | 0.064** | 0.025 | 0.087 |
| Action planning*→*Behavior | 0.203(0.025) | 0.207*** | 0.162 | 0.245 |
| Coping planning*→*Behavior | 0.327(0.023) | 0.391*** | 0.289 | 0.366 |
| Indirect effects |  |  |  |  |
| ASE→Intention, MSE*→*Action planning | 0.092(0.012) | 0.132*** | 0.073 | 0.114 |
| ASE→Intention, MSE*→*Coping planning | 0.076(0.012) | 0.093*** | 0.059 | 0.098 |
| ASE→Intention, MSE→Action planning ,coping planning→Behavior | 0.086(0.011) | 0.126*** | 0.070 | 0.105 |
| Attitude*→*Intention→Action planning | 0.057(0.016) | 0.043*** | 0.035 | 0.087 |
| Attitude*→*Intention→Coping planning | 0.058(0.015) | 0.037*** | 0.035 | 0.085 |
| Attitude*→*Intention→Behavior | 0.018(0.007) | 0.014*** | 0.09 | 0.032 |
| Attitude*→*Intention→Action planning, coping planning*→*Behavior | 0.045(0.012) | 0.034*** | 0.027 | 0.068 |
| Subjective norms*→*Intention*→*Action planning | 0.026(0.012) | 0.025* | 0.008 | 0.047 |
| Subjective norms*→*Intention*→*Coping planning | 0.026(0.011) | 0.022* | 0.009 | 0.046 |
| Subjective norms*→*Intention*→*Behavior | 0.008(0.004) | 0.008* | 0.003 | 0.017 |
| Subjective norms*→*Intention→Action planning, coping planning*→*Behavior | 0.020(0.009) | 0.020* | 0.007 | 0.037 |
| Perceived behavioral control→Intention*→*Action planning | 0.045(0.008) | 0.055*** | 0.032 | 0.060 |
| Perceived behavioral control*→*Intention*→*Coping planning | 0.045(0.010) | 0.047*** | 0.035 | 0.059 |
| Perceived behavioral control*→*Intention*→*Behavior | 0.126(0.013) | 0.158*** | 0.107 | 0.149 |
| Perceived behavioral control*→*Intention→Action planning, coping planning*→*Behavior | 0.128(0.013) | 0.158*** | 0.108 | 0.149 |
| Intention→Action planning, coping planning*→*Behavior | 0.113(0.013) | 0.130*** | 0.092 | 0.136 |
| MSE→Action planning, coping planning*→*Behavior | 0.136(0.016) | 0.132*** | 0.112 | 0.163 |
| Total effects |  |  |  |  |
| ASE*→*Behavior | 0.086(0.011) | 0.126*** | 0.070 | 0.105 |
| Attitude*→*Behavior | 0.045(0.012) | 0.034*** | 0.027 | 0.068 |
| Subjective norms→Behavior | 0.020(0.009) | 0.020* | 0.007 | 0.037 |
| Perceived behavioral control*→*Behavior | 0.176(0.02) | 0.218*** | 0.143 | 0.208 |
| Intention*→*Behavior | 0.168(0.02) | 0.194*** | 0.134 | 0.202 |
| MSE*→*Behavior | 0.286(0.028) | 0.278*** | 0.241 | 0.332 |

*Note.* Age, sex, educational status, and occupational status were included as control variables in the structural equation model. ASE = Action self-efficacy; SN = Subjective Norm; PBC = Perceived behavioral control; MSE = Maintenance self-efficacy; AP = Action planning; CP = Coping planning; B = Unstandardized path coefficient; SE = Standard error; β = Standardized path coefficient; 95% CI = 95% confidence interval of unstandardized path coefficient; LL = Lower limit of 95% CI; UL = Upper limit of 95% CI. ^*^*p* < .05 ^**^*p* < .01 ^***^*p* < .001

.391^***^

.380^***^

.104^***^

.177^***^

.105^*^

.227^***^

.060^*^

.146^***^

.282^***^

.189^***^

.241^***^

.206^***^

.064^**^

.207^***^

*Figure S1*. Standardized path coefficients among constructs from the integrated social cognition model with hand hygiene as the target behavior. Age sex, educational status, and occupational status were control variables in the model. ^*^*p* < .05, ^**^*p* < .01, ^***^*p* < .001

(χ^2^ = 1082.385, *df* = 402; *p* < .001; CFI = 0.964, TLI = 0.956, SRMR = .0810, RMSEA = .040, 90% CI = [.037, .042])

Table S3

*Direct, Indirect, and Total Effects in the Structural Equation Model Testing Relations Among the Integrated Social Cognition Model Constructs with Practicing Respiratory Hygiene as the Target Behavior*

| Path | B (SE) | β | 95% CI | |
| --- | --- | --- | --- | --- |
|  |  |  | LL | UL |
| Direct effects |  |  |  |  |
| ASE*→*Intention | 0.084(0.025) | 0.106** | 0.043 | 0.127 |
| Attitude*→*Intention | 0.271(0.062) | 0.177*** | 0.168 | 0.372 |
| SN*→*Intention | 0.121(0.051) | 0.105* | 0.038 | 0.208 |
| Perceived behavioral control*→*Intention | 0.212(0.026) | 0.227*** | 0.169 | 0.255 |
| Perceived behavioral control*→*Behavior | 0.03(0.018) | 0.037 | 0.00 | 0.058 |
| ASE*→*MSE | 0.254(0.02) | 0.381*** | 0.222 | 0.288 |
| MSE→Action planning | 0.295(0.031) | 0.282*** | 0.243 | 0.347 |
| MSE→Coping planning | 0.231(0.033) | 0.188*** | 0.177 | 0.285 |
| MSE→Behavior | 0.196(0.024) | 0.191*** | 0.157 | 0.234 |
| Intention*→*Action planning | 0.213(0.026) | 0.242*** | 0.171 | 0.256 |
| Intention*→*Coping planning | 0.213(0.026) | 0.207*** | 0.172 | 0.255 |
| Intention*→*Behavior | 0.115(0.018) | 0.134*** | 0.086 | 0.146 |
| Action planning*→*Behavior | 0.207(0.023) | 0.212*** | 0.169 | 0.247 |
| Coping planning*→*Behavior | 0.361(0.022) | 0.431*** | 0.325 | 0.397 |
| Indirect effects |  |  |  |  |
| ASE→Intention, MSE*→*Action planning | 0.093(0.012) | 0.133*** | 0.074 | 0.115 |
| ASE→Intention, MSE*→*Coping planning | 0.077(0.012) | 0.094*** | 0.059 | 0.098 |
| ASE→Intention, MSE→Action planning ,coping planning→Behavior | 0.106(0.012) | 0.156*** | 0.087 | 0.128 |
| Attitude*→*Intention→Action planning | 0.058(0.016) | 0.043*** | 0.035 | 0.087 |
| Attitude*→*Intention→Coping planning | 0.058(0.015) | 0.037*** | 0.035 | 0.085 |
| Attitude*→*Intention→Behavior | 0.037(0.010) | 0.029*** | 0.022 | 0.054 |
| Attitude*→*Intention→Action planning, coping planning*→*Behavior | 0.064(0.016) | 0.049*** | 0.039 | 0.092 |
| Subjective norms*→*Intention*→*Action planning | 0.026(0.012) | 0.025* | 0.008 | 0.047 |
| Subjective norms*→*Intention*→*Coping planning | 0.026(0.011) | 0.022* | 0.009 | 0.046 |
| Subjective norms*→*Intention*→*Behavior | 0.017(0.008) | 0.017* | 0.005 | 0.031 |
| Subjective norms*→*Intention→Action planning, coping planning*→*Behavior | 0.029(0.012) | 0.029* | 0.009 | 0.050 |
| Perceived behavioral control→Intention*→*Action planning | 0.045(0.008) | 0.055*** | 0.032 | 0.060 |
| Perceived behavioral control*→*Intention*→*Coping planning | 0.045(0.007) | 0.047*** | 0.035 | 0.059 |
| Perceived behavioral control*→*Intention*→*Behavior | 0.030(0.005) | 0.038*** | 0.022 | 0.039 |
| Perceived behavioral control*→*Intention→Action planning, coping planning*→*Behavior | 0.152(0.013) | 0.188*** | 0.131 | 0.175 |
| Intention→Action planning, coping planning*→*Behavior | 0.121(0.014) | 0.140*** | 0.100 | 0.145 |
| MSE→Action planning, coping planning*→*Behavior | 0.144(0.017) | 0.141*** | 0.118 | 0.174 |
| Total effects |  |  |  |  |
| ASE*→*Behavior | 0.106(0.012) | 0.156*** | 0.087 | 0.128 |
| Attitude*→*Behavior | 0.064(0.016) | 0.049*** | 0.039 | 0.092 |
| Subjective norms→Behavior | 0.029(0.012) | 0.029*** | 0.009 | 0.050 |
| Perceived behavioral control*→*Behavior | 0.181(0.019) | 0.225*** | 0.150 | 0.212 |
| Intention*→*Behavior | 0.236(0.020) | 0.274*** | 0.203 | 0.269 |
| MSE*→*Behavior | 0.340(0.027) | 0.332*** | 0.296 | 0.384 |

*Note.* Age, sex, educational status, and occupational status were included as control variables in the structural equation model. ASE = Action self-efficacy; SN = Subjective Norm; PBC = Perceived behavioral control; MSE = Maintenance self-efficacy; AP = Action planning; CP = Coping planning; B = Unstandardized path coefficient; SE = Standard error; β = Standardized path coefficient; 95% CI = 95% confidence interval of unstandardized path coefficient; LL = Lower limit of 95% CI; UL = Upper limit of 95% CI. ^*^*p* < .05 ^**^*p* < .01 ^***^*p* < .001

.381^***^

.106^**^

.177^***^

.105^*^

.227^***^

.037

.191^***^

.282^***^

.188^***^

.242^***^

.207^***^

.134^***^

.212^***^

.431^***^

*Figure S2*. Standardized path coefficients among constructs from the integrated social cognition model with practicing respiratory hygiene as the target behavior. Age sex, educational status, and occupational status were control variables in the model. ^*^*p* < .05, ^**^*p* < .01, ^***^*p* < .001

(χ^2^ = 1510.971, *df* = 402; *p* < .001; CFI = 0.964, TLI = 0.955, SRMR = .0808, RMSEA = .040, 90% CI = [.038, .042])

Table S4

*Direct, Indirect, and Total Effects in the Structural Equation Model Testing Relations Among the Integrated Social Cognition Model Constructs with Maintaining a One-Meter Distance as the Target Behavior*

| Path | B (SE) | β | 95% CI | |
| --- | --- | --- | --- | --- |
|  |  |  | LL | UL |
| Direct effects |  |  |  |  |
| ASE*→*Intention | 0.084(0.025) | 0.106** | 0.044 | 0.127 |
| Attitude*→*Intention | 0.270(0.062) | 0.177*** | 0.167 | 0.372 |
| SN*→*Intention | 0.122(0.051) | 0.105* | 0.039 | 0.208 |
| Perceived behavioral control*→*Intention | 0.212(0.026) | 0.227*** | 0.169 | 0.255 |
| Perceived behavioral control*→*Behavior | 0.054(0.017) | 0.070** | 0.026 | 0.084 |
| ASE*→*MSE | 0.255(0.020) | 0.382*** | 0.223 | 0.289 |
| MSE→Action planning | 0.293(0.031) | 0.282*** | 0.242 | 0.345 |
| MSE→Coping planning | 0.232(0.033) | 0.189*** | 0.177 | 0.285 |
| MSE→Behavior | 0.165(0.023) | 0.168*** | 0.127 | 0.204 |
| Intention*→*Action planning | 0.212(0.026) | 0.241*** | 0.169 | 0.254 |
| Intention*→*Coping planning | 0.214(0.026) | 0.207*** | 0.172 | 0.256 |
| Intention*→*Behavior | 0.098(0.017) | 0.119*** | 0.069 | 0.125 |
| Action planning*→*Behavior | 0.181(0.024) | 0.192*** | 0.142 | 0.220 |
| Coping planning*→*Behavior | 0.348(0.021) | 0.435*** | 0.314 | 0.383 |
| Indirect effects |  |  |  |  |
| ASE→Intention, MSE*→*Action planning | 0.092(0.012) | 0.133*** | 0.073 | 0.115 |
| ASE→Intention, MSE*→*Coping planning | 0.077(0.012) | 0.094*** | 0.059 | 0.098 |
| ASE→Intention, MSE→Action planning ,coping planning→Behavior | 0.094(0.011) | 0.143*** | 0.079 | 0.114 |
| Attitude*→*Intention→Action planning | 0.057(0.016) | 0.043*** | 0.035 | 0.086 |
| Attitude*→*Intention→Coping planning | 0.058(0.015) | 0.037*** | 0.035 | 0.085 |
| Attitude*→*Intention→Behavior | 0.037(0.010) | 0.029*** | 0.022 | 0.054 |
| Attitude*→*Intention→Action planning, coping planning*→*Behavior | 0.057(0.014) | 0.045*** | 0.035 | 0.082 |
| Subjective norms*→*Intention*→*Action planning | 0.026(0.012) | 0.025* | 0.035 | 0.047 |
| Subjective norms*→*Intention*→*Coping planning | 0.026(0.011) | 0.022* | 0.035 | 0.046 |
| Subjective norms*→*Intention*→*Behavior | 0.017(0.008) | 0.017* | 0.005 | 0.134 |
| Subjective norms*→*Intention→Action planning, coping planning*→*Behavior | 0.026(0.011) | 0.027* | 0.035 | 0.045 |
| Perceived behavioral control→Intention*→*Action planning | 0.045(0.008) | 0.055*** | 0.032 | 0.060 |
| Perceived behavioral control*→*Intention*→*Coping planning | 0.045(0.007) | 0.047*** | 0.035 | 0.059 |
| Perceived behavioral control*→*Intention*→*Behavior | 0.030(0.005) | 0.038*** | 0.022 | 0.039 |
| Perceived behavioral control*→*Intention→Action planning, coping planning*→*Behavior | 0.143(0.013) | 0.185*** | 0.123 | 0.165 |
| Intention→Action planning, coping planning*→*Behavior | 0.113(0.013) | 0.136*** | 0.092 | 0.136 |
| MSE→Action planning, coping planning*→*Behavior | 0.134(0.016) | 0.136*** | 0.109 | 0.161 |
| Total effects |  |  |  |  |
| ASE*→*Behavior | 0.094(0.011) | 0.143*** | 0.076 | 0.114 |
| Attitude*→*Behavior | 0.057(0.014) | 0.045*** | 0.035 | 0.082 |
| Subjective norms→Behavior | 0.026(0.011) | 0.027* | 0.008 | 0.045 |
| Perceived behavioral control*→*Behavior | 0.197(0.019) | 0.255*** | 0.166 | 0.229 |
| Intention*→*Behavior | 0.211(0.019) | 0.255*** | 0.181 | 0.243 |
| MSE*→*Behavior | 0.299(0.025) | 0.304*** | 0.259 | 0.341 |

*Note.* Age, sex, educational status, and occupational status were included as control variables in the structural equation model. ASE = Action self-efficacy; SN = Subjective Norm; PBC = Perceived behavioral control; MSE = Maintenance self-efficacy; AP = Action planning; CP = Coping planning; B = Unstandardized path coefficient; SE = Standard error; β = Standardized path coefficient; 95% CI = 95% confidence interval of unstandardized path coefficient; LL = Lower limit of 95% CI; UL = Upper limit of 95% CI. ^*^*p* < .05 ^**^*p* < .01 ^***^*p* < .001

.382^***^

.106^**^

.177^***^

.105^***^

.227^***^

.070^**^

.168^***^

.282^***^

.189^***^

.241^***^

.207^***^

.119^***^

.192^***^

.435^***^

*Figure S3*. Standardized path coefficients among constructs from the integrated social cognition model with maintaining a one meter distance as the target behavior. Age sex, educational status, and occupational status were control variables in the model. ^*^*p* < .05, ^**^*p* < .01, ^***^*p* < .001

(χ^2^ = 1508.682, *df* = 402; *p* < .001; CFI = 0.964, TLI = 0.955, SRMR = .0806, RMSEA = .040, 90% CI = [.038, .042])

Table S5

*Direct, Indirect, and Total Effects in the Structural Equation Model Testing Relations Among the Integrated Social Cognition Model Constructs with Staying at Home if Unwell as the Target Behavior*

| Path | B (SE) | β | 95% CI | |
| --- | --- | --- | --- | --- |
|  |  |  | LL | UL |
| Direct effects |  |  |  |  |
| ASE*→*Intention | 0.082(0.025) | 0.104*** | 0.042 | 0.125 |
| Attitude*→*Intention | 0.270(0.062) | 0.176*** | 0.166 | 0.371 |
| SN*→*Intention | 0.121(0.051) | 0.105* | 0.038 | 0.208 |
| Perceived behavioral control*→*Intention | 0.212(0.026) | 0.227*** | 0.169 | 0.255 |
| Perceived behavioral control*→*Behavior | 0.092(0.019) | 0.118*** | 0.061 | 0.122 |
| ASE*→*MSE | 0.252(0.020) | 0.378*** | 0.221 | 0.286 |
| MSE→Action planning | 0.295(0.031) | 0.282*** | 0.244 | 0.347 |
| MSE→Coping planning | 0.232(0.033) | 0.189*** | 0.178 | 0.286 |
| MSE→Behavior | 0.127(0.025) | 0.128*** | 0.086 | 0.169 |
| Intention*→*Action planning | 0.212(0.026) | 0.241*** | 0.169 | 0.255 |
| Intention*→*Coping planning | 0.214(0.026) | 0.206*** | 0.172 | 0.256 |
| Intention*→*Behavior | 0.053(0.019) | 0.064** | 0.022 | 0.083 |
| Action planning*→*Behavior | 0.149(0.025) | 0.158*** | 0.109 | 0.192 |
| Coping planning*→*Behavior | 0.317(0.023) | 0.394*** | 0.280 | 0.355 |
| Indirect effects |  |  |  |  |
| ASE→Intention, MSE*→*Action planning | 0.092(0.012) | 0.132*** | 0.073 | 0.114 |
| ASE→Intention, MSE*→*Coping planning | 0.076(0.012) | 0.093*** | 0.058 | 0.098 |
| ASE→Intention, MSE→Action planning ,coping planning→Behavior | 0.074(0.010) | 0.112*** | 0.058 | 0.091 |
| Attitude*→*Intention→Action planning | 0.057(0.016) | 0.043*** | 0.034 | 0.086 |
| Attitude*→*Intention→Coping planning | 0.058(0.015) | 0.036*** | 0.035 | 0.085 |
| Attitude*→*Intention→Behavior | 0.022(0.007) | 0.017*** | 0.012 | 0.035 |
| Attitude*→*Intention→Action planning, coping planning*→*Behavior | 0.041(0.011) | 0.032*** | 0.025 | 0.061 |
| Subjective norms*→*Intention*→*Action planning | 0.026(0.012) | 0.025* | 0.008 | 0.047 |
| Subjective norms*→*Intention*→*Coping planning | 0.026(0.011) | 0.022* | 0.009 | 0.046 |
| Subjective norms*→*Intention*→*Behavior | 0.010(0.005) | 0.010* | 0.003 | 0.020 |
| Subjective norms*→*Intention→Action planning, coping planning*→*Behavior | 0.018(0.008) | 0.019* | 0.006 | 0.034 |
| Perceived behavioral control→Intention*→*Action planning | 0.045 | 0.055*** | 0.032 | 0.060 |
| Perceived behavioral control*→*Intention*→*Coping planning | 0.045 | 0.047*** | 0.035 | 0.059 |
| Perceived behavioral control*→*Intention*→*Behavior |  |  |  |  |
| Perceived behavioral control*→*Intention→Action planning, coping planning*→*Behavior | 0.122 | 0.157*** | 0.103 | 0.142 |
| Intention→Action planning, coping planning*→*Behavior | 0.099(0.012) | 0.119*** | 0.081 | 0.121 |
| MSE→Action planning, coping planning*→*Behavior | 0.118(0.016) | 0.119*** | 0.093 | 0.145 |
| Total effects |  |  |  |  |
| ASE*→*Behavior | 0.074(0.010) | 0.112*** | 0.058 | 0.091 |
| Attitude*→*Behavior | 0.041(0.011) | 0.032*** | 0.025 | 0.061 |
| Subjective norms→Behavior | 0.018(0.008) | 0.019* | 0.006 | 0.034 |
| Perceived behavioral control*→*Behavior | 0.214(0.019) | 0.275*** | 0.182 | 0.246 |
| Intention*→*Behavior | 0.152(0.020) | 0.183*** | 0.121 | 0.185 |
| MSE*→*Behavior | 0.244(0.026) | 0.247*** | 0.201 | 0.287 |

*Note.* Age, sex, educational status, and occupational status were included as control variables in the structural equation model. ASE = Action self-efficacy; SN = Subjective Norm; PBC = Perceived behavioral control; MSE = Maintenance self-efficacy; AP = Action planning; CP = Coping planning; B = Unstandardized path coefficient; SE = Standard error; β = Standardized path coefficient; 95% CI = 95% confidence interval of unstandardized path coefficient; LL = Lower limit of 95% CI; UL = Upper limit of 95% CI. ^*^*p* < .05 ^**^*p* < .01 ^***^*p* < .001

.378^***^

.104^***^

.176^***^

.105^*^

.227^***^

.118^***^

.128^***^

.282^***^

.189^***^

.241^***^

.206^***^

.064^**^

.158^***^

.394^***^

*Figure S4*. Standardized path coefficients among constructs from the integrated social cognition model with staying at home if unwell as the target behavior. Age sex, educational status, and occupational status were control variables in the model. ^*^*p* < .05, ^**^*p* < .01, ^***^*p* < .001

(χ^2^ = 1426.579, *df* = 402; *p* < .001; CFI = 0.966, TLI = 0.958, SRMR = .0789, RMSEA = .039, 90% CI = [.036, .041])
